# Roxadustat improves diabetic myocardial injury by upregulating HIF-1α/UCP2 against oxidative stress

**DOI:** 10.1186/s12933-025-02601-2

**Published:** 2025-02-07

**Authors:** Tingting Fang, Congcong Ma, Bingyun Yang, Meiyu Zhao, Luning Sun, Ningning Zheng

**Affiliations:** Department of Pathophysiology, College of Basic Medical Science, China Medical University, No.77 Puhe Road, Shenyang North New Area, Shenyang, 110122 Liaoning China

**Keywords:** Roxadustat, Diabetes mellitus, Diabetic myocardial injury, Uncoupling protein 2, Hypoxia-inducible factor -1α, Oxidative stress

## Abstract

**Background:**

Diabetes mellitus (DM), characterized by hyperglycemia, is intricately linked with cardiovascular complications. Hyperglycemia induces oxidative stress, compromising mitochondria energy metabolism disturbances, leading to cardiomyocyte hypoxia and dysregulation of hypoxia-inducible factor-1α (HIF-1α), thereby exacerbating diabetic myocardial injury. Roxadustat (FG-4592), as an inhibitor of HIF-PHD, reduces HIF-1α degradation and regulates the transcription and function of downstream target genes. This study explores the protective effect of FG-4592 on the diabetic myocardium and further investigates the specific mechanisms responsible for this action.

**Methods:**

We established diabetic myocardial injury mice and high glucose-induced rat cardiomyocyte models, administered FG-4592 pretreatment to clarify the protective effects and related mechanisms of FG-4592 on diabetic myocardial injury by detecting changes in oxidative stress, mitochondrial function, and related pathways.

**Results:**

FG-4592 demonstrated cardioprotective effects in diabetic mice by regulating mitochondrial structure and function, as well as maintaining oxidative stress balance in the myocardium. It stabilized HIF-1α, activated UCP2, and enhanced the PI3K/AKT/Nrf2 pathway, reducing mitochondrial superoxide production, improving mitochondrial respiratory potential, and modulating oxidative stress markers in high glucose-induced cardiomyocytes.

**Conclusions:**

FG-4592 exerts protective effects against diabetic myocardial injury by reducing oxidative stress. The mechanism is linked with the upregulation of HIF-1α and UCP2, which subsequently activate the PI3K/AKT/Nrf2 signaling pathway.

**Supplementary Information:**

The online version contains supplementary material available at 10.1186/s12933-025-02601-2.

## Introduction

Diabetes mellitus (DM) is a metabolic disease characterized by persistent hyperglycemia [1]. It is mainly characterized by insufficient insulin production or insulin resistance. According to the latest estimates from the International Diabetes Federation Atlas, the number of people with diabetes is now over 450 million worldwide and is expected to reach 693 million in 2045 [2]. Among the types of diabetes, type 2 diabetes mellitus (T2DM) represents the majority of cases, accounting for more than 90% of individuals with diabetes.

DM and its related complications represent a significant cause of mortality among patients worldwide [3]. Clinical data indicate that approximately 30–40% of patients with diabetes develop at least one complication within 10 years of diagnosis. Once complications arise, they are challenging to reverse with medication, which significantly elevates the mortality rate of diabetic patients. Diabetes-related myocardial injury represents a significant complication that is associated with high mortality in patients. Its pathophysiological mechanisms are complex and regulated by multiple factors. The current literatures indicate that processes such as oxidative stress, disturbed energy metabolism, apoptosis and inflammation are all involved in the development of myocardial injury. Among these, oxidative stress is particularly significant in cellular damage [4–7].

As cellular energy suppliers, it is important to maintain the normal structure and function of mitochondria to maintain cellular homeostasis, especially for cardiomyocytes, which have a high energy demand. In 2014, Montaigne et al. demonstrated the presence of mitochondrial dysfunction in atrial myocytes of patients with T2DM [8]. Furthermore, they showed that the restoration of mitochondrial function alleviated the contractile dysfunction of the myocardium that occurs in Lep^db/db mice [9]. Mitochondria are in turn key organelles for the production of reactive oxygen species (ROS) and the maintenance of oxidative stress homeostasis. Mitochondrial uncoupling proteins (UCPs) are a family of anion transport proteins present in the inner mitochondrial membrane. They are closely associated with changes in energy metabolism, preventing mitochondria from undergoing glucose oxidation and promoting glycolysis through a substrate shunting mechanism. Uncoupling protein 2 (UCP2), a member of UCPs family, plays an important role in reducing mitochondrial ROS production and protecting cells from oxidative stress damage [10]. Meanwhile, it has been shown that resveratrol can play a protective role against diabetic cardiomyopathy by increasing the expression of UCP2 to reduce the generation of ROS [11].

The PI3K/AKT signaling pathway plays a pivotal role in regulating cardiomyocyte morphology, survival, apoptosis, protein synthesis and metabolism. These processes are essential for maintaining the normal physiological function of the heart [12]. Insulin can promote glucose uptake through the activation of PI3K, thereby facilitating the recovery of postischemic function in rat hearts [13]. Furthermore, it can ameliorate oxidative stress injury in H9c2 cells induced by microRNA-1 through the activation of AKT [14]. Nuclear factor erythroid lineage 2-related factor 2 (Nrf2) is a pivotal regulator of the antioxidant stress system. Liao et al. demonstrated that activation of Nrf2 through the PI3K/AKT signaling pathway significantly enhanced the antioxidant capacity of hepatocytes [15]. Hesperidin also attenuated high-fat diet-induced oxidative stress injury in rat hepatocytes by activating the PI3K/AKT/Nrf2 pathway [16]. It was shown that Nrf2 also indirectly down-regulates the gene activity of UCP2 via miR-195 and miR-497 [17]. As a key regulatory pathway and regulator of cellular resistance to oxidative stress, the interaction between PI3K/AKT/Nrf2 and UCP2 is essential for maintaining oxidative stress homeostasis.

The stress produced by hyperglycemia on mitochondria causes disorders in mitochondrial energy metabolism, leaving tissues and cells in a relatively hypoxic environment for a prolonged period of time. In diabetic tissues, despite the severity of hypoxia, the stability and function of HIF-1 is inhibited, resulting in insufficient activation of HIF-1, thus impairing the adaptive response to hypoxia, and the resulting pathological changes are the underlying pathogenic factors in the development of diabetes and diabetic complications [18]. HIF-1 is a heterodimeric transcription factor consisting of two subunits, HIF-1α and HIF-1β [19], which are widely expressed in mammalian cells. In the normoxic conditions, the prolyl hydroxylase domain (PHD) of HIF binds to HIF-1α, promoting its degradation. In hypoxic conditions, a reduction in hydroxylase activity results in the stabilization of HIF-1α protein, which is then translocated to the nucleus and binds to HIF-1β, thereby activating its transcriptional activity. This permits the cellular transcriptional response to low oxygen levels [20]. It has been shown that increased expression of HIF-1α in mouse kidney epithelial cells under sustained hyperglycaemic conditions can reduce their mitochondrial ROS overproduction and exert a protective effect. FG-4592 is a novel oral anti-renal anemia drug that was approved for marketing in China in December 2018 [21]. As an inhibitor of HIF-PHD, FG-4592 reversibly binds to it, inhibiting the degradation of HIF-1α by reducing its activity. This promotes the entry of HIF-1α into the nucleus, where it activates the transcriptional activity of its downstream pathway [22–23]. Studies have demonstrated that FG-4592 protects cardiomyocytes from adriamycin (DOX)-induced apoptosis and mitochondrial ROS production by increasing the expression of HIF-1α, the apoptosis inhibitory protein Bcl-2, and the antioxidant enzyme SOD2 [24]. FG-4592 also enhances the expression of SOD2 and heme oxygenase-1 (HO-1) in endotoxin-induced H9c2 cells, thereby protecting mitochondria through antioxidant effects [25].

However, the specific role and mechanism of FG-4592 in diabetic myocardial injury have not yet been reported in studies. The aim of this study was to test the hypothesis that FG-4592 can exert an anti-oxidative stress effect through the upregulation of HIF-1α/UCP2 and protect against diabetic myocardial injury.

##  Materials and methods

### Animals

38 male C57BL/6J mice, aged 6–8 weeks, were purchased from Beijing Sipeifu Biotechnology Company. The mice were acclimatized and fed for one week, and then randomly divided into control (*n* = 8) and diabetic groups (*n* = 30). The control group was fed with normal chow (#D12492, Sipeifu Biotechnology Company, Beijing, China), while the diabetic group was fed with a high-fat chow (#SPF-F02-001, Sipeifu Biotechnology Company, Beijing, China) for 8 weeks. Then the diabetic groups randomly divided into Vehicle + T2DM group (*n* = 10) and FG-4592 (#HY-13426, MCE, Shanghai, China) pretreated group (*n* = 20). The FG-4592 pretreated group mice were continuous gavaged for 9 days with two dosage: 10 mg/kg and 25 mg/kg, which were defined as FG10 + T2DM group (*n* = 10) and FG25 + T2DM group (*n* = 10), respectively. And the mice in the Vehicle + T2DM group were treated with an equal volume of double-distilled water by gavage for 9 days. At the end of the gavage, mice in these three groups were injected intraperitoneally with streptozotocin (40 mg/kg, once a day)(#18883-66-4, SIGMA, USA) for 2–5 days to destroy the pancreatic islets to accelerate diabetes modeling. Diabetic mice were confirmed by blood glucose (fasting blood glucose > 11.1 mmol/L) and body weight, then euthanized immediately. All animals were housed in standard, plastic rodent cages, under controlled conditions, with a constant temperature of 24 ± 1 ^◦^C and a 12-h light/12-h dark cycle (lights on at 07:00 h) and ad libitum access to food and water.

###  Body weight, blood pressure, and blood glucose measurements in mice

Body weight measurement: Ensure the scale (#ZK-DST2, Zhike, Henan) is clean and calibrated. Then, gently place the mouse on the scale. Allow the mouse to settle and stabilize on the scale. Once the mouse is stationary, immediately record the body weight displayed on the scale. Blood pressure measurement: Carefully place the mouse on the Softron sphygmomanometer (#BP-98 A, Softron Bio Tech Co. Ltd., Beijing, China) and position the blood pressure sensor as close as possible to the anterior end of the mouse’s tail. Allow the mouse to relax and its heart rate to stabilize. Once the heart rate is stable, record the blood pressure reading displayed on the sphygmomanometer. Blood Glucose Measurement: Carefully restrain the mouse and stabilize its body. Gently snip the end of the mouse’s tail to initiate blood flow. Wipe away the first drop of blood with a paper towel. Collect the second drop of blood and carefully place it on the Roche Glucose Meter (Accu-chek, Roche, Irish). Record the blood glucose value displayed on the meter. Body weight, blood glucose and blood pressure were recorded every fortnight.

###  Myocardial tissue specimen collection and processing

After all the experiments were completed, mice were anaesthetized by intraperitoneal injection of 1% pentobarbital (45 mg/kg)(#P11011, Merck, USA), then blood was collected from the eyeballs to collect the serum of the mice, and the thoracic cavities were dissected under a dissecting microscope rapidly to expose the heart to a good range of operation, and the heart was irrigated with physiological saline to exclude the blood that might be in the heart and removed immediately after irrigation. The mouse heart was removed immediately after the perfusion was completed. After 1 mm^3^ of myocardial tissue was fixed in 2.5% glutaraldehyde (#111-30-8, Merck, USA) for 24 h, images were collected by transmission electron microscopy(#H-7650, Hitachi Limited, Japan), half of the remaining cardiac tissue was fixed in 4% paraformaldehyde(#BL593A, Biosharp, China) for subsequent morphological experiments, and the rest was stored in a refrigerator (#702, Thermo, USA) at -80 ^◦^C for subsequent molecular biology experiments.

###  Echocardiographic measurements

Left ventricular (LV) function was assessed by a fijifilm visualsonic 3100LT echo-cardiographic system (Fujifilm Visual Sonics, China) at a probe frequency of 10 MHz with M-mode recording. Mice were anesthetized with avertin and depilated with depilating cream on the chest. The limbs of mice were attached to heating pads to maintain body temperature at 37 ^◦^C. Medical ultrasound gel (Tianjin Yajie Medical Material Co. Ltd., Tianjin, China) was used as a coupling agent between the ultrasound scan-head and the skin. Left ventricular parameters such as Left Ventricular Ejection Fraction and Left Ventricular Fraction Shortening were measured by M-mode recording. The data are presented as the average of measurements of three consecutive beats.

### Transmission electron microscopy (TEM)

Myocardial tissues were collected and fixed by 2.5% glutaraldehyde (#111-30-8,Merck, USA). After being washed in 0.1 M sodium cacodylate buffer (#18839, Merck, USA), tissues were postfixed with 1% buffered osmium (20816-12-0, Merck, USA). The tissues were dehydrated through the graded alcohol (#64-17-5, Merck, USA) and embedded in resin (#JM3852, HPBIO, Shanghai, China). After dehydration through graded alcohol and embedding in resin, tissues were incubated in a 60 ^◦^C oven for 3 days. Subsequently examined by a transmission electron microscope (#H-7650, Hitachi Limited, Japan).

### Brain natriuretic peptide (BNP) detection assay

Brain Natriuretic Peptide (BNP) was measured in serum from mice treated with different treatments using a Brain Natriuretic Peptide assay kit (#E-EL-M0204c, Elabscience, Wuhan, China) according to the manufacturer’s instructions and quantified with absorbance at 450 nm on a microplate reader (#A51119700DPC, Thermo Fisher, USA).

###  Cell culture and treatment

The rat heart-derived cardiac cells H9c2 (H9c2 cells) were purchased from Shanghai Cell Bank, Chinese Academy of Sciences. H9c2 cells were cultured in DMEM (#SH30022.01, HyClone, Shanghai, China) medium with 10% FBS (#164210-50, Procell, Wuhan, China), 1% penicillin and streptomycin antibiotics (#PB180120, Procell, Wuhan, China). Cells were incubated in an atmosphere of 5% CO_2_ at 37 ˚C. When the cells reached 50% confluency, H9c2 cells were pretreated with FG-4592 (10 mM) for 24 h and then stimulated with glucose (45 mM)(#ab147494, Procell, Wuhan, China) for additional 24 h. After these treatments, cells were harvested for appropriate analyses.

### Western blot assay

Briefly, protein was extracted from tissue samples using RIPA lysis buffer (#P0013B, Beyotime, Shanghai, China) supplemented with protease inhibitors (#ST506-2, Beyotime, Shanghai, China). The protein concentrations in the resulting lysates were determined using a BCA kit (#23225, Thermo Fisher Scientific). For traditional western blot procedure, the following primary antibodies were used: anti-HIF-1α (1:500, #36169S), anti-NRF2 (1:500, #D1Z9C), anti-UCP2 (1:500, #D1O5V), anti-HO-1 antibody (1:500, #70081S), anti-COX4 (1:1,000, #4850S) (all from Cell Signaling Technology, Danvers, Massachusetts, USA), anti-β-actin (1:1,000, #20536-1-AP, Proteintech, Wuhan, China), anti-SOD2 (1:500, #WL02506), anti-AKT (1:500, #WL0003b), anti-P-AKT (1:500, #WLP001a), anti-PI3K (1:500, #WL03380), anti-Bax (1:500,#WL01637) (all from Wanlei Technology, Shenyang, China), anti-P-PI3K (1:500, #AF3242, Affinity, Biosciences, Jiangshu, China), anti-GADPH (1:5,000, #ab9484, Abcam, United States). The membranes were then incubated with appropriate secondary horseradish peroxidase-conjugated anti-rabbit or anti-goat immunoglobulin G (IgG) antibodies at a 1:5000 dilution for 45 min at 37^◦^C. Blots were visualized using an enhanced chemoluminescence (ECL) reagent (#P0018AS, Beyotime, China) and quantified by scanning densitometry (Tanon-5200T, Tanon, Shanghai, China).

###  MDA, GSH, SOD detection

Malondialdehyde (MDA) levels in myocardial tissues under different conditions were measured by the thiobarbituric acid method (#BC0025, Solarbio, Beijing, China). GSH levels (#BC1175, Solarbio, Beijing, China) were measured in tissue samples using a GSH/GSSG assay kit. SOD levels (#BC0175, Solarbio, Beijing, China) were also measured in tissue samples using a SOD assay kit.

### Mitochondrial complexes I and IV and ATP content detection

The activities of the mitochondrial complex I and IV were determined by the Micro Mitochondrial Respiratory Chain Complex I and IV Activity Assay Kit (#BC0515, #BC0945, Solarbio, Beijing, China) according to the manufacturer’s instructions. Briefly, mitochondrial homogenates were added into the respective reaction buffer. The reaction mixture was transferred to a prewarmed (30 °C) quartz cuvette and immediately put into a spectrophotometer. The absorbance of reaction mixture was measured at 340 nm for Complex I, or 550 nm for Complex IV, respectively. Mitochondrial complex activity was expressed as nmol/min/mg protein. ATP was quantified using an ATP assay kit (#S0026, Beyotime, Shanghai, China) according to the manufacturer’s protocols on a microplate reader.

### Cell counting Kit-8 (CCK-8) assay

The H9c2 cell were seeded in 96-well plates, approximately 3,000 cells were seeded per well. After culturing for 24 h at 37 ^◦^C in 5% CO_2_, glucose at different concentrations were added to the cells, each group had at least 3 repetitions. After 24 h, 10uL of CCK-8 reagent (#C0037, Beyotime, Shanghai, China) was added to each well and incubated for 1 h under the above conditions. The absorbance at 450 nm was measured by a microplate reader (PerkinElmer, Wellesley, Massachusetts, USA).

###  OCR measurements

Experiments were performed using 300,000 H9c2 cells per chamber. And mitochondrial function was determined using high-resolution respirometry (Oxygraph 2k, Oroboros, Austria). The analysis was blinded. Briefly, respirometry was performed in respiration medium containing EGTA (0.5 mM), MgCl_2_ (3 mM), K-lactobionate (60 mM), taurine (20 mM), KH_2_PO_4_ (10 mM), HEPES (20 mM), sucrose (110 mM), and fatty-acid-free BSA (1 g/L). OCR was determined by adding oligomycin (#O4876, Oroboros, Austria), proximity compound carbonyl cyanide 4-(trifluoromethoxy) phenylhydrazone (FCCP)(#C2759, Oroboros, Austria), and antimycin A (#A8674, Oroboros, Austria) treatments. OCR (pmol/min) was calculated automatically via Oxygraph 2k.

### Quantitative real-time PCR (qRT-PCR)

Total RNA was extracted using RNAiso Reagent (#9109, TaKaRa Bio, Tokyo, Japan) and reverse-transcribed into cDNA using PrimeScript RT Master Mix (#RR047A, TaKaRa Bio). qRT-PCR was performed using TB Green qPCR Master Mix (#RR420A, TaKaRa Bio). The sequences of the primers used were, HIF-1α: 5′-ACCGCCACCACCACTGATG-3′ and 5′-GTACCACTGTATGCTGATGCCTTAG-3′;GAPDH:5′-GACATGCCGCCTGGAGAAAC-3′ and 5′-AGCCCAGGATGCCCTTTAGT-3′; UCP2: 5′-CTGGCGGTGGTCGGAGATAC-3′ and 5′-TGGCATTTCGGGCAACATTGG-3′.

### Synthesis and transfection of overexpression plasmids and siRNAs

The siRNA of UCP2, HIF-1α and a negative control was designed by Huatuobio (Shenyang, China). DMEM (#SH30022.01, HyClone, Shanghai, China) was used to dilute transfection reagent (#KC112-1.5, Huatuobio, Shenyang, China) and siRNA, respectively. After 5 min, the two were mixed, and the mixture was added to serum-free cell culture medium 20 minutes later. The sequences of siRNAs for rat are in Supplementary Table 1.

### Mitosox red staining

After 24 hours’ exposure to FG-4592 and 45 mM glucose levels in normoxia, H9c2 cells were stained with MitoSOX™Red Mitochondrial Superoxide Indicator (#M36008, Thermo Fisher Scientific, Germany). A working concentration of 500nM was used, and cells were incubated at 37 ^◦^C for 30 minutes protected from light. After washing off excess dye, the cell crawls were placed under a confocal microscope (Nikon AXR, Japan) for observation and photography.

### Immunohistochemical (IHC) staining

For IHC studies, paraffin-embedded cardio sections were deparaffi-nized and hydrated using slide warmers and alcohol. After antigen re-trieval, the sections were permeabilized with 3% H_2_O_2_ and blocked with 5% bovine serum albumin (BSA). Then, the sections were individually incubated with the following antibodies at appropriate concentrations: antibody against HIF-1α. Then, the sections were incubated with secondary antibodies and reacted with diaminobenzidine (DAB) in accordance with the manufacturer’s instructions.

### Bioinformatics analysis of RNA-seq

Fastp software (https://github.com/OpenGene/fastp) were used to remove the reads that contained adaptor contamination, low quality bases and undetermined bases with default parameter. Then sequence quality was also verified using fastp. We used HISAT2 (https://ccb.jhu.edu/software/hisat2) to map reads to the reference genome of RAT. The mapped reads of each sample were assembled using String Tie(https://ccb.jhu.edu/software/stringtie) with default parameters. Then, all transcriptomes from all samples were merged to reconstruct a comprehensive transcriptome using gffcompare(https://github.com/gpertea/gffcompare/). After the final transcriptome was generated, StringTie and was used to estimate the expression levels of all transcripts. StringTie was used to perform expression level for mRNAs by calculating FPKM (FPKM = [total_exon_fragments / mapped_reads (millions) × exon_length(kB) ] ). The differentially expressed mRNAs were selected with fold change > 2 or fold change < 0.5 and with parametric F-test comparing nested linear models (pvalue < 0.05) by R package edgeR (https://bioconductor.org/packages/release/bioc/html/edgeR.html).

### Statistical analysis

All experiments were conducted as biological replicates, with sample size determined by statistical power requirements. Data are expressed as mean ± standard deviation. All analyses were carried out using GraphPad Prism version 8.0 (GraphPad Software, San Diego, CA, USA). Comparisons between groups were made using a t-test (two-sided). One-way ANOVA test were used for comparison of data from multiple groups, respectively. *p* < 0.05 was considered statistically significant.

## Results

###  Construction of a mouse model of T2DM combined with myocardial injury

**Fig. 1:**
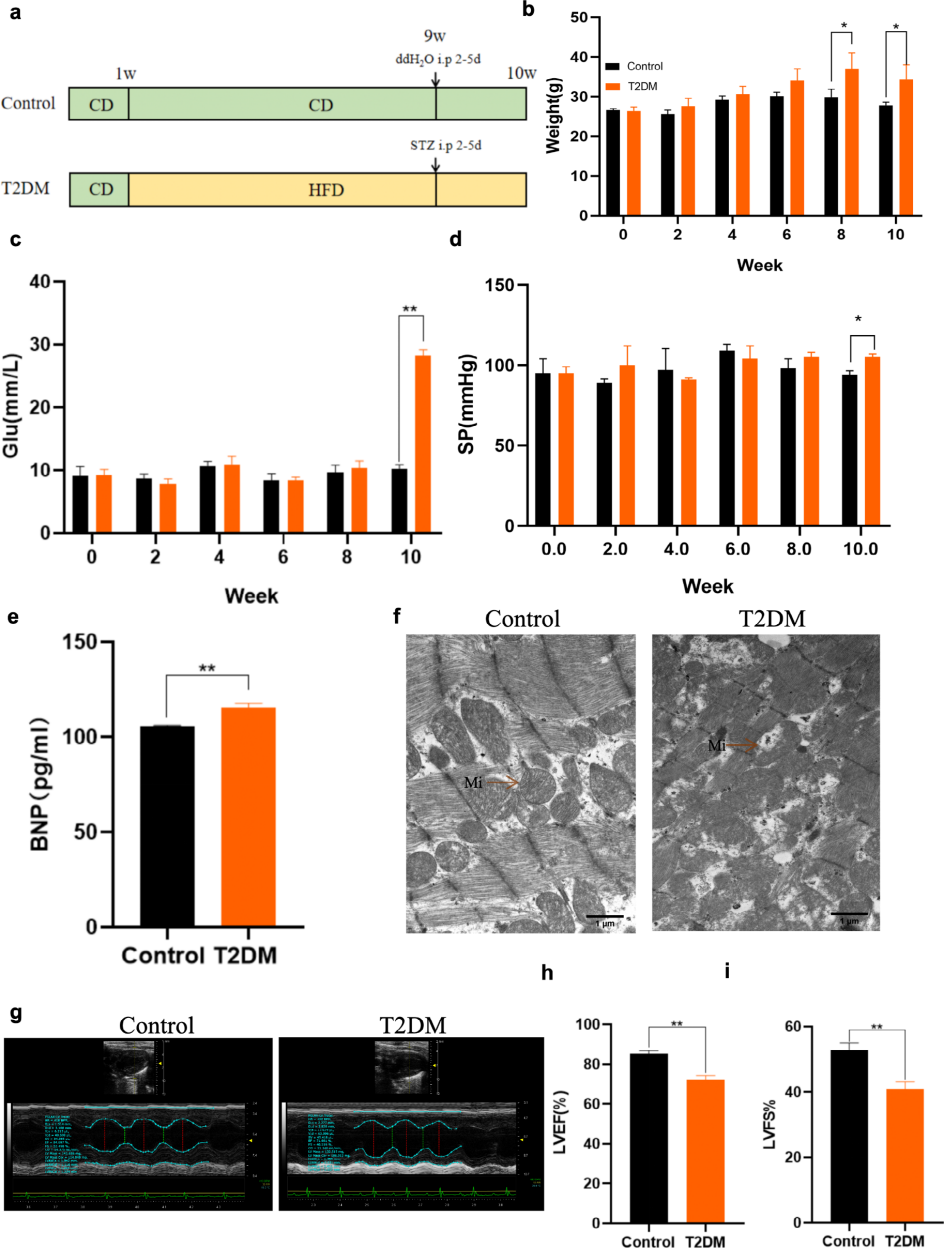
Construction of a mouse model of T2DM combined with myocardial injury. **a **Flowchart of mouse modeling. **b–c** Changes in body weight and blood glucose in control and diabetic mice at weeks 0–10. *n* = 5–6 mice per group. **d** Changes in blood pressure in control and diabetic mice at weeks 0–10. *n* = 5–6 mice per group. **e** Serum BNP levels in control and diabetic mice at week 10. *n* = 5 mice per group. **f** Mitochondrial structure of mouse cardiac muscle tissues under transmission electron microscopy; magnification 20000X, scale bar = 1 μm. *n* = 3 mice per group. **g–i** Changes in LVEF and LVFS, indicators of cardiac function in mice. *n* = 3 mice per group. P values were determined by t-test. **P < 0.01, *P < 0.05

Male C57BL/6J mice (38), aged 6–8 weeks, were divided into control group (*n* = 8) and diabetic group (*n* = 30). The control mice were fed a standard diet, while the diabetic mice were fed a diet consisting of 60% fat for 8 weeks followed by intraperitoneal injection of STZ (40 mg/kg, once daily) over 2–5 days (Fig. 1a). During the modeling period, mice were tested for body weight, blood glucose, and blood pressure every fortnight, and blood glucose levels were measured every day after the beginning of STZ injection. During the modeling period, the control and diabetic mice maintained a continuous increase in body weight. By the 10th week, the diabetic mice exhibited significantly elevated body weight and blood glucose levels compared to the control mice (Fig. 1b–c), indicating the successful establishment of the diabetic mouse model.

At week 10, the diabetic group displayed higher blood pressure and serum B-type natriuretic peptide (BNP) levels (a marker of myocardial injury) than the control group (Fig. 1d–e). In addition, the results of echocardiography indicated that the left ventricular ejection fraction (LVEF) and left ventricular short-axis shortening rate (LVFS) in the diabetic group were reduced (Fig. 1g–i). Examination of myocardial tissue via transmission electron microscopy revealed considerable mitochondrial damage in the diabetic mice, characterized by disorganized, swollen, and irregular mitochondria, disrupted cristae, and vacuoles (Fig. 1f). These observations collectively support the establishment of a mouse model of T2DM associated with myocardial injury.

###  FG-4592 pretreatment improves myocardial injury in T2DM mice

**Fig. 2:**
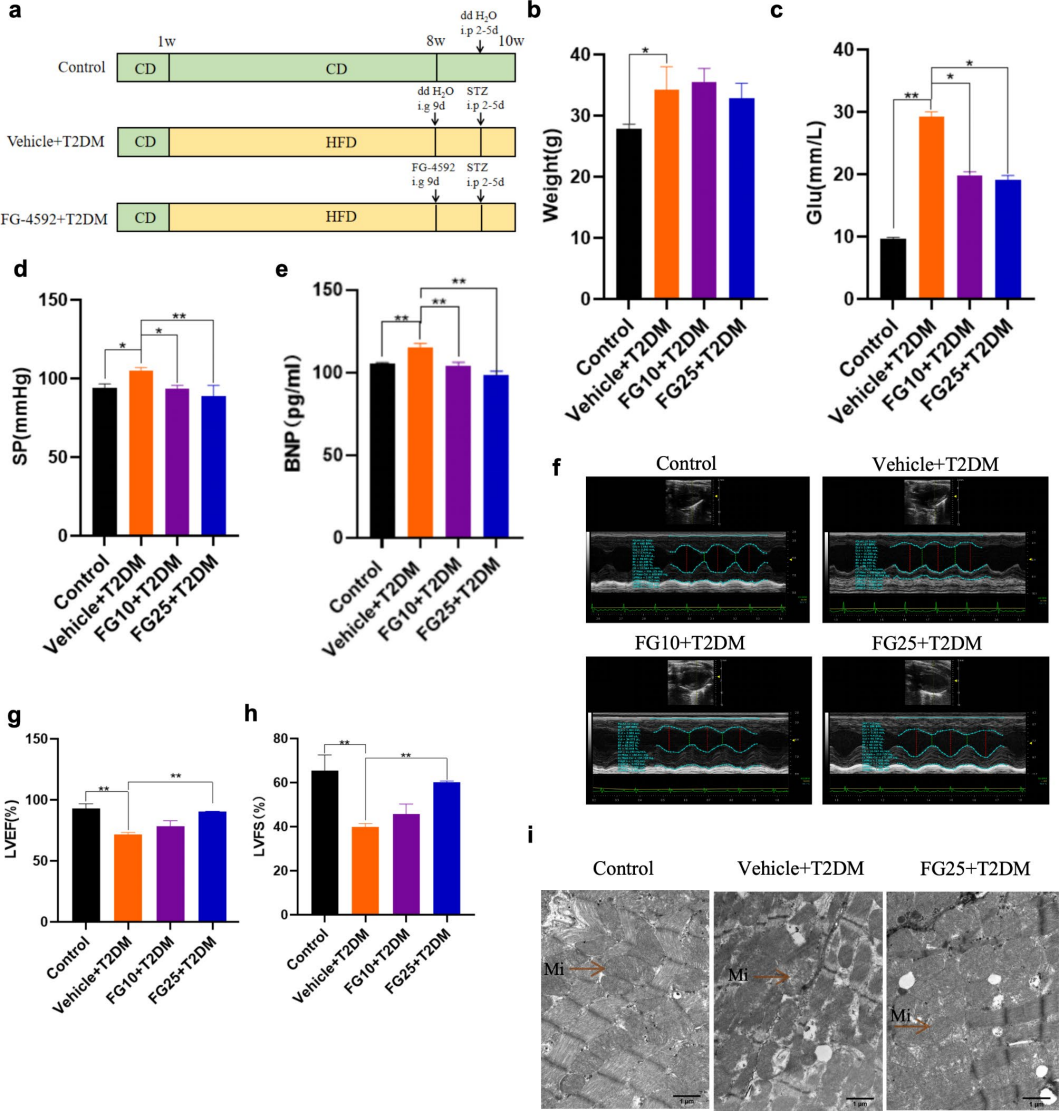
FG-4592 pretreatment improves myocardial injury in T2DM mice. **a **FG-4592 pretreatment modeling process. **b–c** Changes in body weight and blood glucose of mice at week 10. *n* = 6 mice per group. **d** Changes in blood pressure of mice at week 10. *n* = 5–6 mice per group. **e** Serum BNP level of mice at week 10. *n* = 5 mice per group. **f–h** Results of echocardiography of mice as well as the index of LVEF and LVFS Changes. *n* = 3 mice per group. **i** Mitochondrial structure of mouse cardiac muscle tissues under transmission electron microscopy; magnification 20000X, scale bar = 1 μm. *n* = 3 mice per group. P values were determined by one-way ANOVA. **P < 0.01, *P < 0.05

To evaluate the effect of FG-4592 on myocardial injury in T2DM mice, we selected mice exhibiting T2DM combined with myocardial injury as our experimental subjects. Firstly, FG-4592 was pre-treated by gavage for 9 days at concentrations of 10 mg/kg/day and 25 mg/kg/day, and then followed by STZ intraperitoneal injection for 2–5 days. After successful modeling, mice were categorized into the following four groups: Control, Vehicle + T2DM, FG10 (FG-4592, 10 mg/kg) + T2DM and FG25 (FG-4592, 25 mg/kg) + T2DM groups (Fig. 2a). Comparative analysis revealed significant reductions in blood glucose levels in FG10 + T2DM and FG25 + T2DM groups compared with Vehicle + T2DM group individually. However, no significant weight loss was observed (Fig. 2b–c). The blood pressure and serum BNP level, indicated reductions in FG10 + T2DM and FG25 + T2DM groups compared with Vehicle + T2DM group individually (Fig. 2d–e). Echocardiographic results showed that only FG-4592 (25 mg/kg) pretreatment improved LVEF and LVFS levels (Fig. 2f–h). The effect of the FG-4592 (10 mg/kg) was not statistically significant. Combined with the changes in the above relevant indicators, we selected FG-4592 (25 mg/kg) as the drug concentration for subsequent in vivo experiments. Electron microscopy results revealed that structural damage, mitochondrial disarrangement, swelling, irregularity, cristae breakage and vacuole-like changes in cardiomyocytes were ameliorated in the FG25 + T2DM group (Fig. 2i). Collectively, these results indicate that FG-4592 pretreatment could improve the cardiac function of T2DM mice and demonstrated notable myocardial protective effects.

### FG-4592 pretreatment attenuates oxidative stress and improves mitochondrial respiratory efficiency in myocardial tissue of diabetic mice

**Fig. 3:**
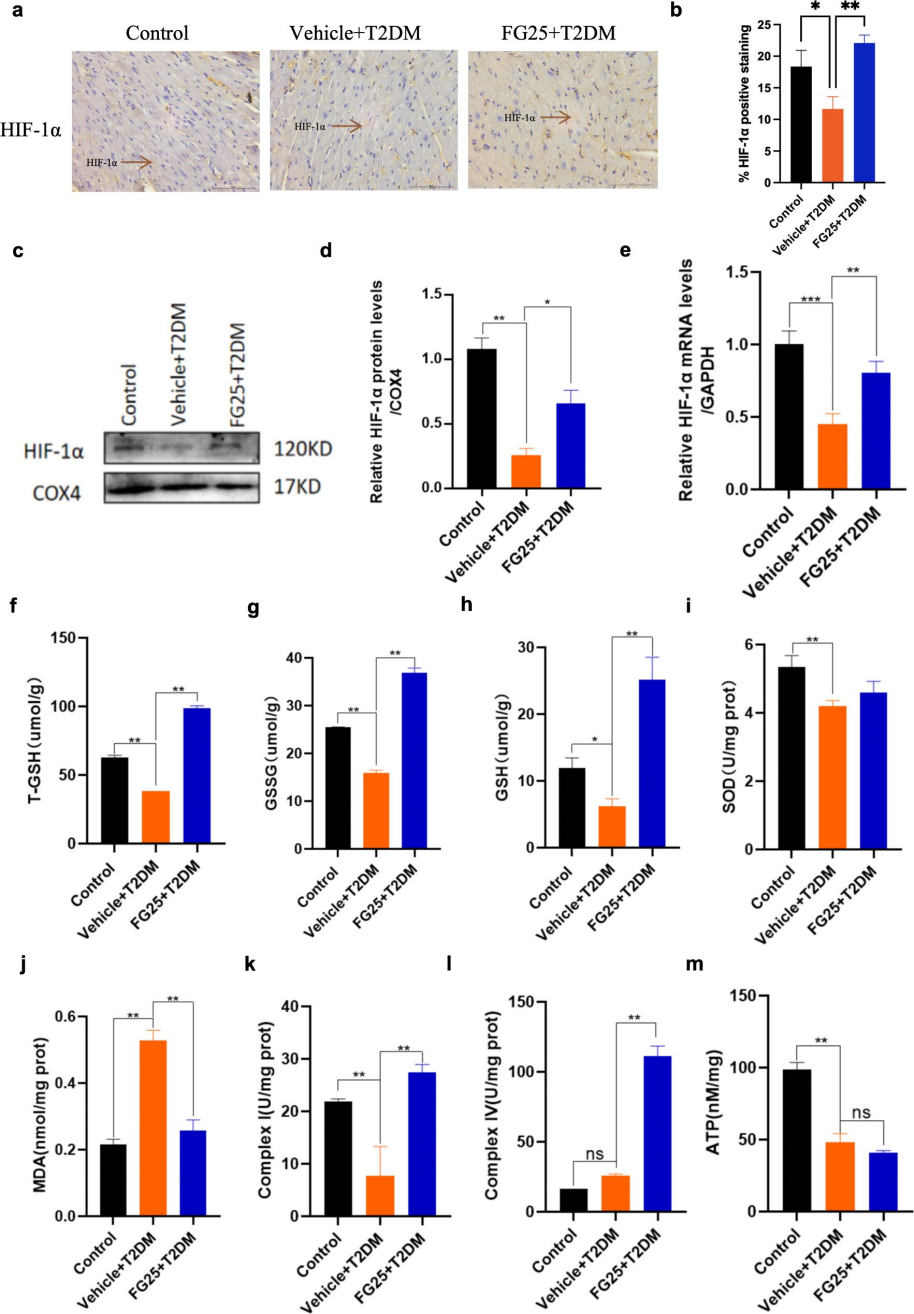
FG-4592 pretreatment attenuates oxidative stress injury and regulates mitochondrial respiratory efficiency in myocardial tissue of diabetic mice. **a-b** HIF-1α expression detected by immunohistochemistry in myocardial tissues, magnification 400x, scale bar = 50 μm. *n* = 5 mice per group. **c–d** HIF-1α expression detected by Western Blot. *n* = 5 mice per group. **e** HIF-1α mRNA level in mice detected by RT-qPCR. *n* = 4 mice per group. **f–h** Expression of T-GSH, GSSH and GSH in myocardial tissues. *n* = 4 mice per group. **i** Expression of superoxide dismutase expression. *n* = 5 mice per group. **j** MDA expression in myocardial tissues. *n* = 4 mice per group. **k–l** Mitochondrial complex I and IV activities in myocardial tissues. *n* = 5 mice per group. **m** Mitochondrial ATP levels in myocardial tissues. *n* = 5 mice per group. P values were determined by one-way ANOVA. ***P < 0.001, **P < 0.01, *P < 0.05, ns represents no statistical significance

Oxidative stress stands as a pivotal mechanism in the development of DM. To further explore how FG-4592 exerts a protective effect on diabetic myocardium, we scrutinized alterations in oxidative stress and mitochondrial respiration-related parameters in myocardial tissues across experimental groups. Initially, the expression of HIF-1α in myocardial tissues of mice in each group was detected by immunohistochemistry, Western Blot and RT-qPCR. The results unveiled a significant reduction in HIF-1α expression in the T2DM group, subsequently restored with FG-4592 pretreatment, indicating FG-4592’s ability to stabilize HIF-1α expression in the of T2DM mice under relative hypoxia conditions (Fig. 3a–e). Subsequently, we assessed oxidative stress-related indexes. Relative to the control group, myocardial tissue levels of total glutathione (T-GSH), oxidized glutathione (GSSG), reduced glutathione (GSH), and superoxide dismutase (SOD) were significantly diminished in the myocardium of the T2DM group. T-GSH, GSSG and GSH were significantly elevated after pretreatment with FG-4592 (Fig. 3f–i). Conversely, the level of lipid peroxides (MDA) significantly increased in the T2DM group, followed by a decrease after FG-4592 pretreatment (Fig. 3j). Given that ROS-mediated oxidative stress damage can impair the function of mitochondria, which in turn interferes with mitochondrial respiration, and we further examined the activity of mitochondrial complex I, complex IV, and the production of ATP to assess mitochondria respiratory efficiency. The results showed that the activities of complex I and complex IV were significantly enhanced in the FG-4592 pretreatment group compared to the T2DM group (Fig. 3k–i). ATP synthesis exhibited a significant reduction in the T2DM group compared with the control group, but no significant changes were observed after FG-4592 pretreatment (Fig. 3m). These findings collectively suggest that FG-4592 can counteract myocardial oxidative stress injury by modulating the balance between oxidation and antioxidants, while also regulating mitochondrial respiratory chain function and efficiency, thereby safeguarding against diabetic myocardial damage.

### Transcriptome sequencing screening for differentially expressed genes and functional enrichment analysis to identify differential gene UCP2 and PI3K/AKT enrichment pathway

**Fig. 4:**
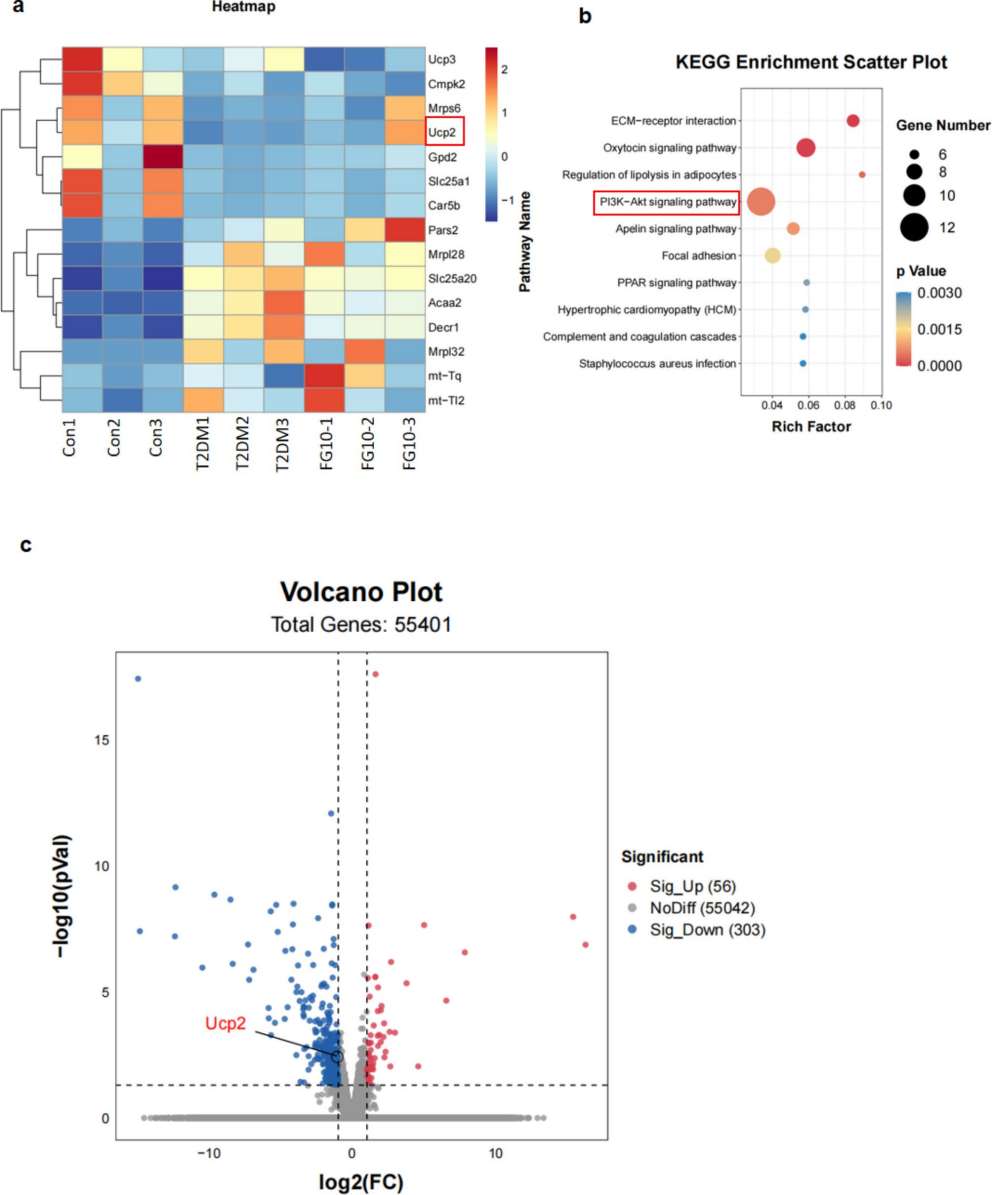
Transcriptome sequencing screening for differentially expressed genes and functional enrichment analysis. **a** Heatmap of transcriptome sequencing screening for genes related to mitochondria. **b** KEGG enrichment analysis of gene expression data. **c** Volcano map of differentially expressed genes, blue represents down-regulation and red represents up-regulation. *n* = 3 mice per group

To further explore the pivotal molecules and related mechanisms by which FG-4592 up-regulates HIF-1α to exert anti-oxidative stress, we conducted transcriptome sequencing of myocardial tissues from mice across three groups, followed by differential gene expression analysis and functional enrichment assessment. The sequencing results unveiled 56 up-regulated and 303 down-regulated genes in the T2DM group compared to the FG-4592 pretreatment group. Notably, among these genes, the expression of mitochondrial uncoupling protein UCP2, which is closely related to cellular oxidative stress, exhibited a decrease in the T2DM group and an increase in the FG-4592 group. In addition, through KEGG enrichment analysis of gene expression data from both T2DM and FG-4592 pretreatment groups, we identified several signal transduction pathways and metabolic pathways were enriched in the FG-4592 group. Particularly, the PI3K/AKT signaling pathway, intricately involved in cell growth, metabolism, and oxidative stress, displayed substantial enrichment. This observation strongly suggests the PI3K/AKT pathway may play a key role in the anti-oxidative stress of FG-4592, thereby underscoring its pivotal role in counteracting oxidative stress (Fig. 4a–c).

### FG-4592 upregulates HIF-1α as well as UCP2 expression in high glucose-induced H9c2 cardiomyocytes

**Fig. 5:**
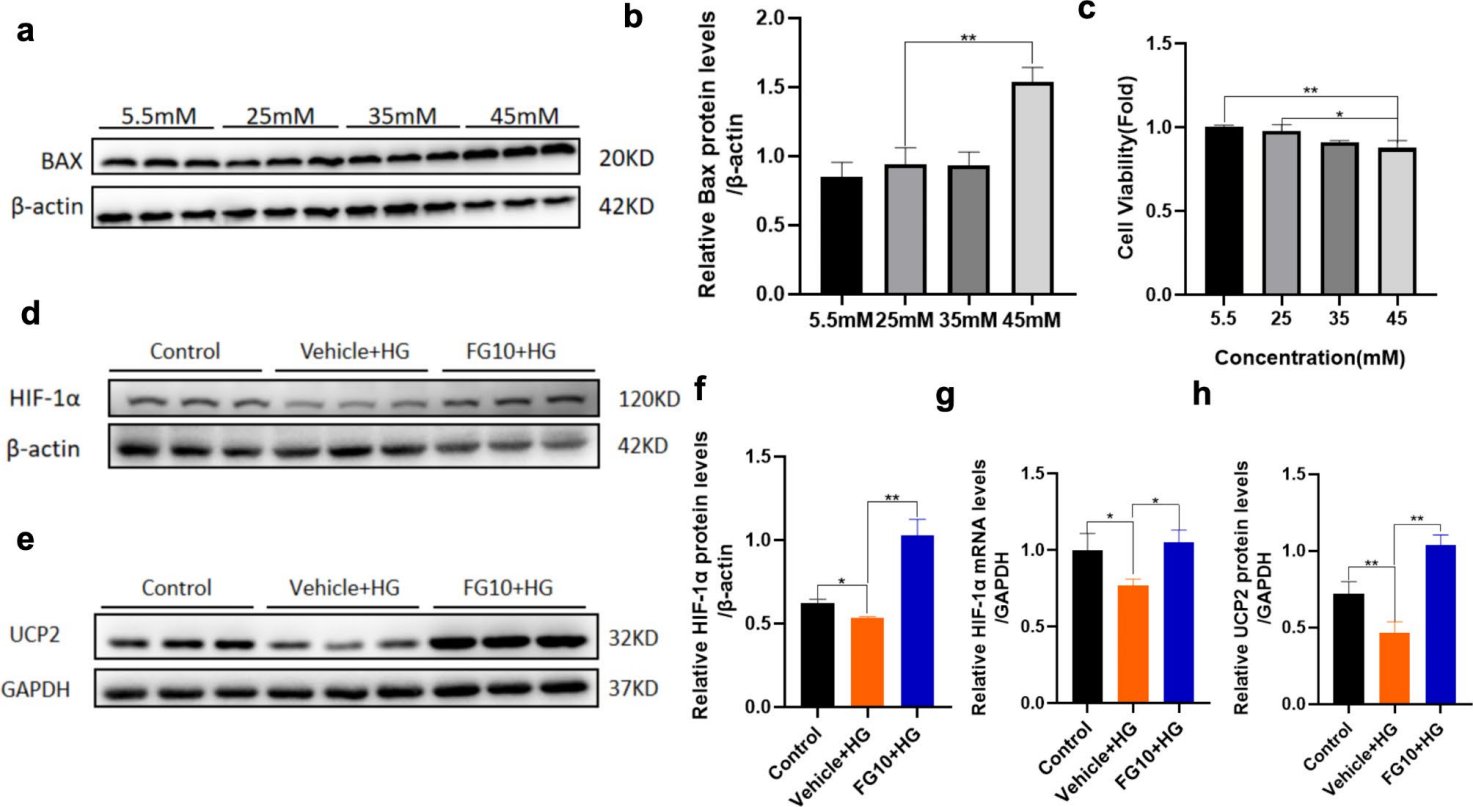
Expression of HIF-1α and UCP2 in high sugar-induced H9c2 cardiomyocyte model. **a-b** Expression of Bax at different sugar concentrations. *n* = 3 experiments per group. **c** CCK8 detection of the effect of different sugar concentrations on the proliferation of H9c2 cells. *n* = 4 experiments per group. **d–e** Western Blot detection of the expression of HIF-1α and UCP2 in H9c2 cells. *n* = 3 experiments per group. **f** Quantitative statistical results of HIF-1α Western Blot in H9c2 cells. **g** RT-qPCR detection of HIF-1α mRNA level in H9c2 cells. *n* = 3 experiments per group. **h** Quantitative statistical results of Western Blot for UCP2. P values were determined by one-way ANOVA. **P < 0.01, *P < 0.05

Building upon the insights gleaned from transcriptome sequencing, we identified the differentially expressed gene UCP2 as a focal point for further investigation. Subsequently, we embarked on a comprehensive exploration of FG-4592’s anti-oxidative stress mechanism through the establishment of cell models. Firstly, we constructed a high glucose-induced cardiomyocyte model. H9c2 cells cultured in DMEM with 45 mM glucose were validated as a high glucose model by the results of CCK8 cell proliferation assay and pro-apoptotic indicator Bax (Fig. 5a–c). Next, the cells were divided into three groups: Control (25 mM), Vehicle + HG (45 mM), and FG10 + HG (FG-4592 10 µM + 45 mM) groups. Evaluation of HIF-1α and UCP2 expression in H9c2 cells via Western Blot and RT-qPCR corroborated the findings from both animal experiments and transcriptome sequencing: In the HG group, the expression of HIF-1α and UCP2 was decreased, whereas pretreatment with FG-4592 led to a significant upregulation of both. These results suggested that UCP2 might be a critical target for FG-4592 to up-regulate the anti-oxidative stress effect of HIF-1α (Fig. 5d–h).

### FG-4592 combats oxidative stress and enhances mitochondrial respiratory potential by upregulating HIF-1α and UCP2

**Fig. 6:**
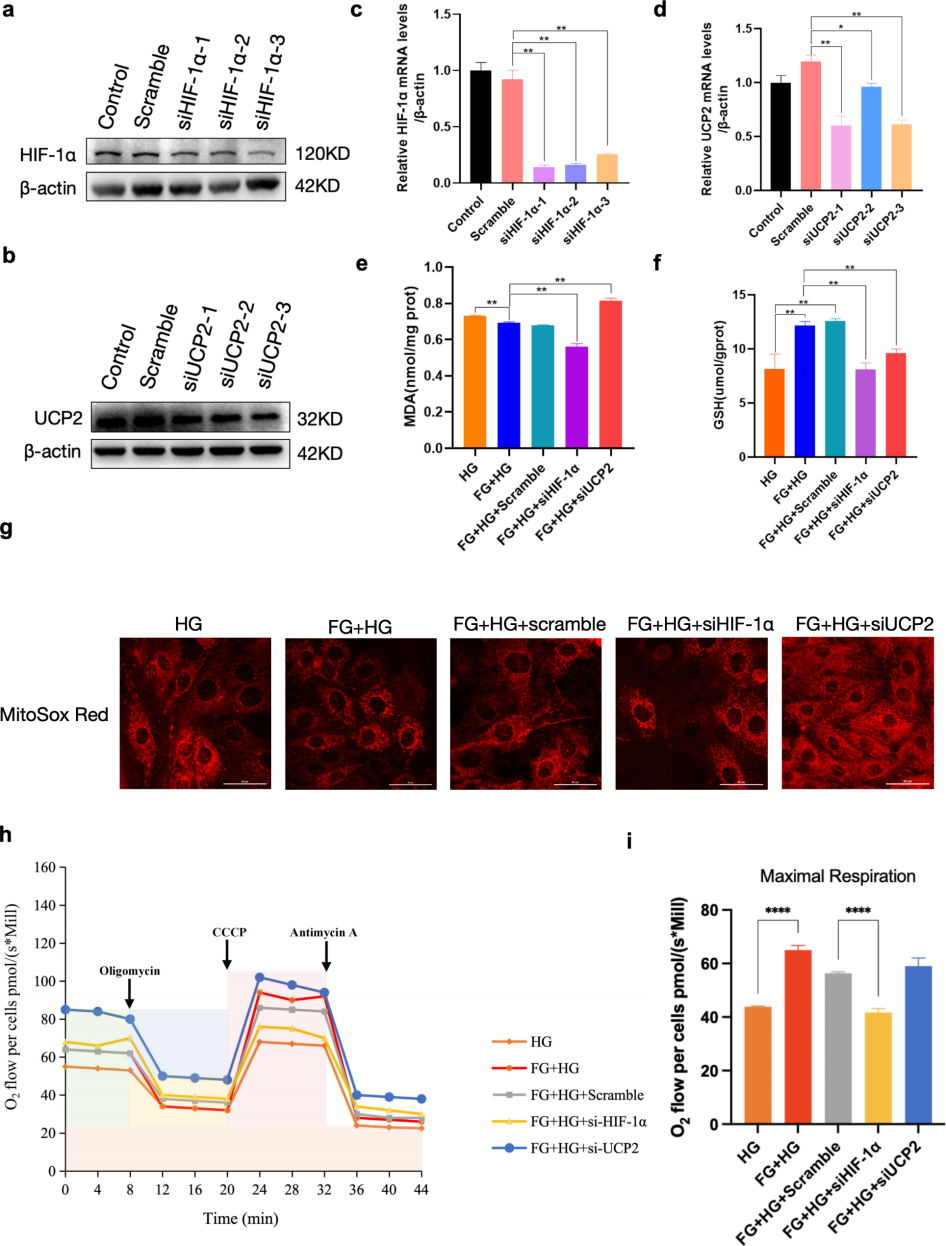
FG-4592 pretreatment improves high glucose-induced oxidative stress and mitochondrial respiratory function in H9c2 cells. **a-d** siRNA knockdown of HIF-1α and UCP2 expression. *n* = 3 experiments per group. **e** MDA content assay. *n* = 4 experiments per group. **f** GSH content assay. *n* = 4 experiments per group. **g** MitoSox Red staining to detect mitochondrial superoxide level, scale bar = 50 μm. *n* = 3 experiments per group. **h–i** O2k to detect mitochondrial oxygen consumption. *n* = 3 experiments per group. P values were determined by one-way ANOVA. **P < 0.01, *P < 0.05

To elucidate further the anti-oxidative stress effects of FG-4592 via upregulation of HIF-1α and UCP2 in cellular models, we employed siRNA to knock down the expression of HIF-1α and UCP2 in high glucose-induced H9c2 cells, respectively (Fig. 6a-d). We selected Hif-1α(Rat)-1 and Ucp2(Rat)-1 in the Supplementary Table 1 for subsequent experiments. The cells were categorized into five groups: HG, FG + HG, FG + HG + scramble, FG + HG + siHIF-1α and FG + HG + siUCP2 groups. And the MDA and GSH levels of these five groups of cells were examined. The results revealed that FG-4592 significantly boosted the level of GSH, indicative of enhanced resistance to oxidative stress, while significantly reducing MDA levels. Conversely, after the knockdown of HIF-1α and UCP2, the protective effects of FG-4592 were diminished, as evidenced by decreased GSH levels (Fig. 6e-f). Additionally, mitochondrial superoxide staining with MitoSox Red demonstrated that FG-4592 pretreatment curtailed superoxide generation under high glucose conditions. Moreover, the superoxide levels were reduced following knockdown of HIF-1α and UCP2, respectively (Fig. 6g). The above results underscored the role of FG-4592 in exerting anti-oxidative stress effects through the upregulation of HIF-1α and UCP2.

O2k technology is a powerful tool for detecting oxygen consumption and mitochondrial function. The technology uses a high-resolution oxygen sensor and a high-sensitivity data acquisition system to monitor changes in oxygen concentration, mitochondrial respiration rate in real-time, and control the mitochondrial respiration process using respiratory chain substrates or inhibitors to provide accurate and reliable mitochondrial respiration analysis. Through oxygen consumption rate (OCR) analysis on five groups of cells, we observed maximum mitochondrial respiration induced by CCCP action in the FG-4592 group compared with the HG group was heightened, suggesting a stronger respiratory reserve capacity. Notably, knockdown of HIF-1α led to a decrease in maximum mitochondrial respiration (Fig. 6h-i). These results implies that FG-4592 may improve mitochondrial respiratory efficiency to a certain extent, thereby supporting cellular adaptation to metabolic stress.

### FG-4592 upregulates HIF-1α and UCP2 against oxidative stress by affecting the PI3K/AKT/Nrf2 pathway

**Fig. 7:**
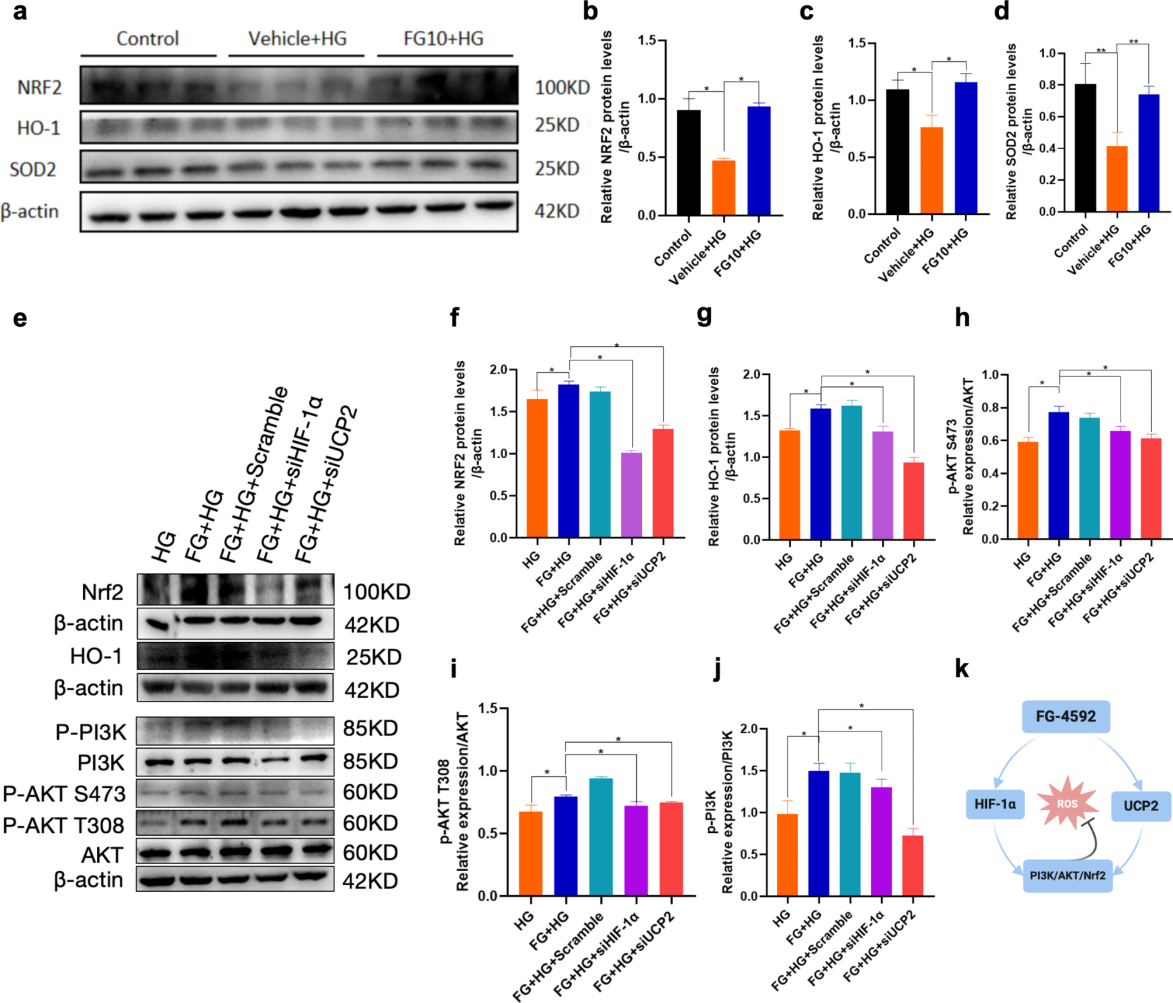
Up-regulation of HIF-1α and UCP2 by FG-4592 pretreatment affects PI3K/AKT/Nrf2 pathway. **a-j** Western Blot for expression of Nrf2, HO-1, SOD2, PI3K, P-PI3K, AKT, P-AKT. *n* = 3 experiments per group. **k** The mechanism cascade of FG-4592 against oxidant stress. Figure created with BioRender.com. *P* values were determined by one-way ANOVA. **P < 0.01, *P < 0.05

The above results showed that FG-4592 could reduce the degree of mitochondrial oxidative stress in cardiomyocytes under high glucose conditions by upregulating the expression of HIF-1α and UCP2. To further investigate the underlying signaling pathway, we detected the levels of Nrf2, HO-1, and SOD2, key anti-oxidative stress-related indexes, using Western Blot analysis across the five groups. Results indicated diminished expression of these markers in the high glucose model group, whereas FG-4592 pretreatment notably elevated their levels, suggesting that FG-4592 can regulate the expression of Nrf2 and HO-1, which were attenuated after the knockdown of HIF-1α and UCP2, respectively (Fig. 7a–g). Notably, the Nrf2 pathway has been shown to affect mitochondrial oxidative stress and potentially interact with UCP2. Based on the suggestion of previous transcriptome sequencing results, we further examined the phosphorylation activation of PI3K and AKT in these five groups, and the results showed that compared with the high-glucose group, revealing significant enhancement following FG-4592 pretreatment. However, this effect was weakened after knockdown of HIF-1α and UCP2, respectively (Fig. 7e, h–j). These results suggest that FG-4592 stabilizes HIF-1α and UCP2 and then activates the PI3K/AKT pathway. This cascade promotes antioxidant responses through Nrf2 and reduces oxidative stress (Fig. 7k).

## Discussion

Our study aimed to explore FG-4592’s potential as an anti-oxidative stress agent to improve diabetic myocardial injury by upregulating HIF-1α and UCP2 expression. Firstly, the efficacy of FG-4592 pretreatment was validated in a mouse model of diabetes combined with myocardial injury. In the subsequent phase of the study, we sought to gain a deeper understanding of the mechanisms by which FG-4592 exerts cardioprotective effects in diabetic myocardiocyte model. A substantial body of evidence indicates that mitochondrial dysfunction and oxidative stress play a pivotal role in the pathogenesis of diabetic cardiac dysfunction. Mitochondrial ultrastructural defects have been observed in mouse cardiomyocytes in a type 1 diabetes model (OVE26) [28]. In models of type 2 diabetes (Lep^db/db and ob/ob), myocardial tissues exhibit significantly reduced mitochondrial ATP synthesis, heightened oxidative stress, elevated ROS production, and structural damage [29–30]. Besides, matrine incubation alleviated DOX-induced H9c2 cells apoptosis and oxidative stress level via activating AMPKα/UCP2 [31]. GPER attenuates cardiac dysfunction in postmenopausal diabetic rats by activating the SIRT1/3-AMPK-UCP2 pathway to exert anti-oxidative stress effects [32]. The excessive production of mitochondrial ROS is not only due to increased electron transport chain activity induced by hyperglycemia but also linked to impaired HIF-1α signaling. Enhanced HIF-1α activity has been demonstrated to suppress ROS production in both in vitro and in vivo diabetic models, thereby mitigating oxidative stress-induced renal injury [33]. Additionally, FG-4592 has been reported to increase the expression of the antioxidant enzyme SOD2 in endotoxin-induced cardiomyocytes, which enhances antioxidant effects and protects mitochondria [25]. Based on these findings, we postulated that FG-4592 could exert a cardioprotective effect in diabetes by reducing oxidative stress injury through the enhancement of HIF-1α and UCP2 expression.

To test this hypothesis, we conducted a series of assays and found that FG-4592 significantly augmented HIF-1α expression in the myocardial tissues of diabetic mice. This confirmed the pharmacological efficacy of FG-4592 in our animal model. Notably, in the T2DM group, anti-oxidative stress indices (T-GSH, GSSH, and GSH) were significantly diminished, while the oxidative stress index (MDA) was markedly increased, indicating a substantial oxidative-antioxidant imbalance in diabetic myocardial tissues. In contrast, the FG-4592 pretreatment group exhibited significantly enhanced antioxidant effects, accompanied by reduced oxidative stress-induced lipid peroxidation damage.

Oxidative stress not only directly harms organisms, but also disrupts the mitochondrial electron transport chain, affecting ATP synthesis and thus mitochondrial respiratory efficiency. Mitochondrial respiratory efficiency is inextricably linked to complex activity and ATP generation. Research has been shown that the mitochondrial respiratory potential of FG-4592-treated dopaminergic neurons was enhanced [34]. Additionally, in diabetic nephropathic rats, FG-4592 significantly improved the activity of mitochondrial complexes I, III, and IV, thus ameliorating mitochondrial dysfunction in diabetic nephropathy [35]. In an ischemic cardiomyocyte model pre-treated with FG-4592, the compound enhances anaerobic glycolysis and maintains ATP production under anaerobic conditions, significantly attenuating myocardial ischemia-reperfusion injury in mice [36]. Our results also demonstrated that FG-4592 pretreatment significantly elevated the activity of myocardial mitochondrial complexes I and IV in diabetic mice. This should contribute to the recovery of mitochondrial respiratory function.

UCP2 emerges as a promising therapeutic target for various vascular diseases, including atherosclerosis, diabetic vascular disease, hypertension, stroke, and peripheral vascular disease [37]. It has been shown that UCP2 alleviates endothelial cell oxidative stress under high glucose induction [38]. In the case of diabetes, UCP2 protects pancreatic β-cells from oxidative stress and glucotoxicity [39]. Here, we demonstrated that FG-4592 reduced ROS levels in H9c2 cells under hyperglycemic conditions, accompanied by high expression of UCP2. It is notable that the anti-oxidative stress effect of FG-4592 was significantly suppressed after knockdown of HIF-1α and UCP2 respectively. These findings call to our attention the crucial role of HIF-1α and UCP2 in FG-4592 cardioprotection. It has also been shown that FG-4592 ameliorates mitochondrial dysfunction in mouse neuroblastoma cells under α-Syn (α-synuclein)-induced mitochondrial dysfunction by increasing mitochondrial basal respiration, ATP production, respiratory reserve, and maximal respiration [40]. Similarly, in our study, FG-4592 significantly enhanced maximal respiration in H9c2 cells under high glucose induction, while these effects were reversed upon knockdown of HIF-1α, consistent with earlier findings.

**Fig. 8:**
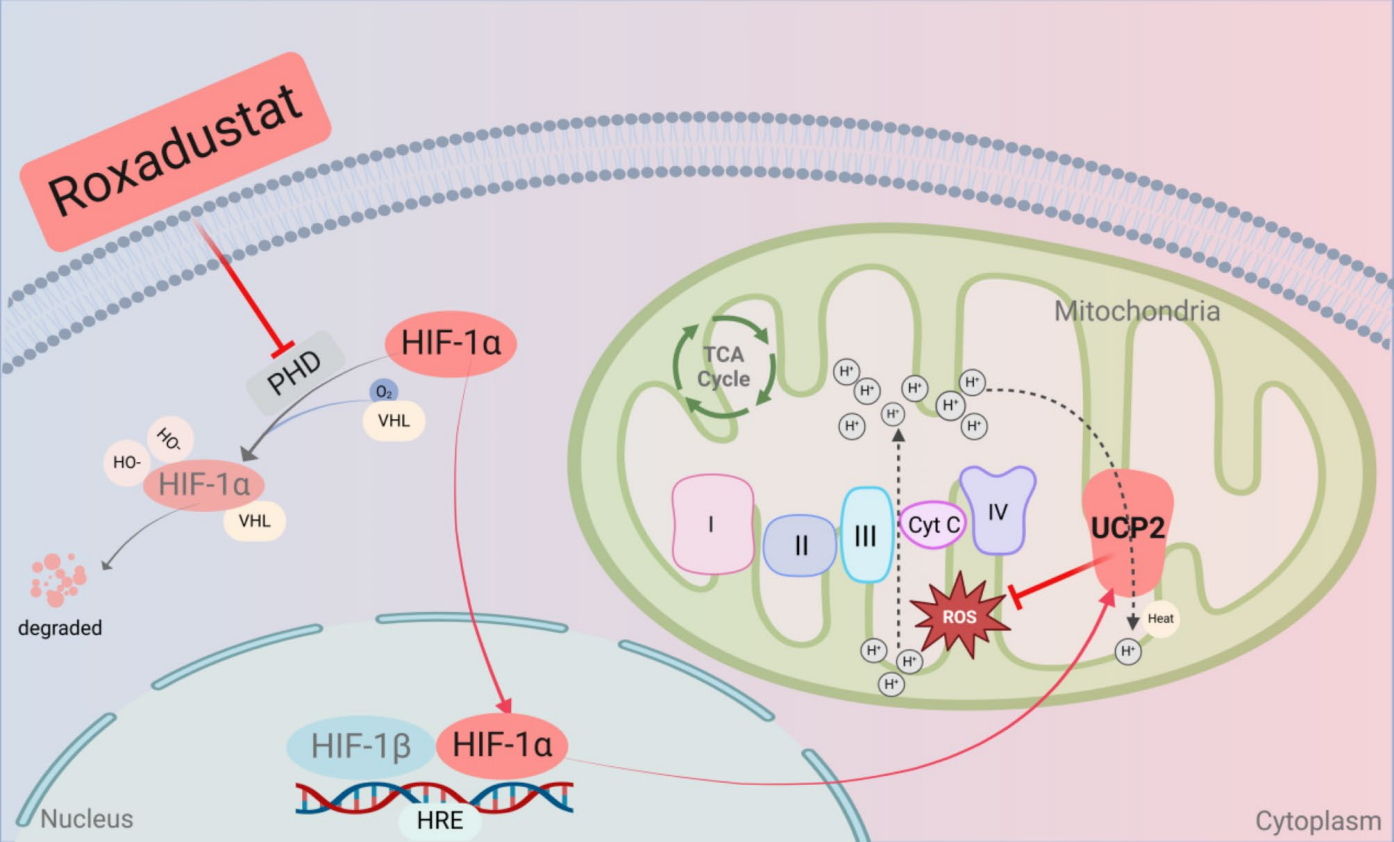
Roxadustat Improves Diabetic Myocardial Injury by Upregulating HIF-1α/UCP2 Against Oxidative Stress. Roxadustat improves diabetic myocardial injury by inhibiting PHD activity increasing the expression of HIF-1α and UCP2, then reduces ROS production in mitochondria. Figure created with BioRender.com

The findings above naturally led us to further investigate the mechanism through which FG-4592 upregulates HIF-1α/UCP2 and then regulates oxidative stress and restores mitochondrial function. Transcriptome results indicated a notable upregulation of genes enriched in the PI3K/AKT pathway in the FG-4592 preconditioned group as compared with the diabetic group. Considering that PI3K/AKT is a pivotal pathway not only for regulating cell survival, migration and proliferation but also for managing oxidative stress. And it has been shown that AKT facilitates the nuclear translocation of Nrf2 and regulates the expression of its downstream antioxidant enzymes [41–42]. Under normal conditions, Nrf2 is sequestered in the cytoplasm by Kelch-like ECH-associated protein 1(Keap1), but during oxidative stress, Nrf2 dissociates from Keap1, translocates to the nucleus, and binds to antioxidant response elements (ARE), thereby activating the transcription of antioxidant genes, including HO-1. HO-1, in turn, mitigates cellular oxidative stress by initiating the breakdown of heme into carbon monoxide and bilirubin [43]. It has been shown that FG-4592 attenuates iron death and thus ameliorates folate-induced renal injury by activating the expression of Nrf2 [44]. Nrf2 regulates the expression of the antioxidant enzymes SOD, GPX, and HO-1, and protects against oxidative stress in hepatocytes [45]. Additionally, FG-4592 induces the expression of HO-1 in mouse neuroblastoma cells, reduces the production of ROS and inhibits α-Syn-induced neurotoxicity [38]. In our study, we found that HO-1, SOD2 and Nrf2 appeared to be significantly decreased in high glucose-induced H9c2 cells, which reconfirmed the enhanced myocardial oxidative stress injury under high glucose conditions. However, the expression of all of these indexes appeared to be significantly increased after FG-4592 pretreatment. The PI3K/AKT/Nrf2 pathway was activated by FG-4592 pretreatment and inhibited by HIF-1α and UCP2 knocking down, respectively. These findings indicate that PI3K/AKT/Nrf2 pathway may be involved in the HIF-1α/UCP2-mediated myocardial protection. In conclusion, the present study demonstrated that FG-4592 exerts a protective effect against diabetic myocardial injury by enhancing the anti-oxidative stress response (Fig. 8).

### Limitations

Our study preliminarily explored the diabetic cardioprotective effects of FG-4592. However, there are still some limitations in this experiment, which we aim to address in future studies. Firstly, our future research will consider investigating intermediate doses, such as 15 mg/kg or 20 mg/kg, to further explore the dose-response relationship in greater detail. This approach could provide a more comprehensive understanding of the optimal dosing strategy and its therapeutic window. Secondly, cardiomyocyte apoptosis is a key component in the development of myocardial injuries, in the aspect of determining whether mice have developed myocardial injury, we should also use more myocardial injury-related indicators for further confirmation, such as the detection of apoptosis-related indicators. Thirdly, we selected COX4 as the housekeeping gene based on its stable expression in animal experiments. However, there are certain limitations to using COX4 as an internal reference. The expression of COX4 may be regulated under specific experimental conditions or in certain tissues, which could affect its reliability as a reference gene. And the expression levels of COX4 may change under specific metabolic states or stress conditions, potentially introducing data bias. Therefore, while its stability was validated in this study, future research should also focus on validating more universally applicable housekeeping genes to further enhance the stability of experimental outcomes. Fourthly, among the MDA experiments, knockdown of HIF-1α did not lead to an increase in MDA levels, which considering that the transfection may play some side effects on cells, which may lead to cell stress response, thus affecting downstream functions. In addition, transfection may trigger non-specific gene expression changes by affecting the HIF-1α level and its associated signaling pathways. These factors may contribute the change of MDA not as expected.

In this study, FG-4592 could exert anti-oxidative stress by improving the expression of HIF-1α and UCP2, but the interaction between HIF-1α and UCP2 was not illuminated clearly, and the transcriptional regulation as well as protein interactions between them were not further described. Undeniably, in addition to the limitations in our study described above, the application of FG-4592 for the treatment of diabetic cardiovascular disease may face some potential encounterings like diabetic retinal damage due to its angiogenesis-promoting effect.

### Future directions

Based on our previous findings, future research will focus on the following directions to further elucidate the mechanisms of FG-4592 and expand its clinical application potential. First, elucidating the regulatory relationship between HIF-1α and UCP2 will be critical to understanding the precise mechanisms by which FG-4592 exerts its cardioprotective effects through antioxidative stress. Secondly, the mechanisms of myocardial metabolic remodelling will be a key focus. As the most mitochondria-rich tissue, the myocardium undergoes significant metabolic changes in metabolic diseases, where dysregulated oxygen sensing pathways and HIF-1α expression contribute to metabolic dysfunction and myocardial injury. Investigating how FG-4592 regulates mitochondrial energy metabolism to improve cardiac function will be a key research priority. In addition, a comprehensive evaluation of mitochondrial functions, including metabolic regulation, mitochondrial homeostasis and inflammatory responses, will be undertaken. This will help to establish a complete mechanistic framework for the cardioprotective effects of FG-4592 through stabilisation of HIF-1α and validate its applicability in different models of metabolic disease. These studies will provide scientific evidence and theoretical support for the development of FG-4592 as a potential cardioprotective drug.

## Conclusion

Our study reveals that FG-4592 exerts protective effects on diabetic myocardial injury in mice and high glucose-induced cardiomyocytes by reducing oxidative stress and enhancing mitochondrial function. This is achieved through the upregulation of HIF-1α and UCP2, which subsequently activates the PI3K/AKT/Nrf2 signaling pathway. These findings offer a novel perspective for exploring the mechanisms underlying diabetic myocardial injury and propose the basic experimental evidence to broaden the application of FG-4592 in managing diabetic heart disease.

## Electronic supplementary material

Below is the link to the electronic supplementary material.

Supplementary Material 1.

## Data Availability

No datasets were generated or analysed during the current study.
