# Supplementary material for: Roxadustat improves diabetic myocardial injury by upregulating HIF-1α/UCP2 against oxidative stress
Source: Cardiovasc Diabetol. 2025 Feb 7;24:67. doi: 10.1186/s12933-025-02601-2 (PMC11806548; doi:10.1186/s12933-025-02601-2)
Supplement: Supplementary file 1 — Supplementary Material 1. [file 12933_2025_2601_MOESM1_ESM.docx]

Supplementary Table 1

|  | sense strand | antisense strand |  |
| --- | --- | --- | --- |
| Hif1a(Rat)-1 | GCUCACCAUCAGUUACUUATT | UAAGUAACUGAUGGUGAGCTT | Selected |
| Hif1a(Rat)-2 | GCCAGCAAGUCCUUCUGAUTT | AUCAGAAGGACUUGCUGGCTT |  |
| Hif1a(Rat)-3 | CCAAGGAGCCUUAACCUAUTT | AUAGGUUAAGGCUCCUUGGTT |  |
| Ucp2(Rat)-1 | GGCCUCUACGACUCUGUAATT | UUACAGAGUCGUAGAGGCCTT | Selected |
| Ucp2(Rat)-2 | CCCGAAAUGCCAUUGUCAATT | UUGACAAUGGCAUUUCGGGTT |  |
| Ucp2(Rat)-3 | GGAUCCUGGAACGUAGUAATT | UUACUACGUUCCAGGAUCCTT |  |
